# Impact of Individualized Education on Mothers’ Knowledge and Practices in Providing Care for Preterm Babies in Uttarakhand

**DOI:** 10.7759/cureus.73872

**Published:** 2024-11-17

**Authors:** Suman Rikhari, Rajkumari Sylvia Devi, Vandana Chauhan

**Affiliations:** 1 Child Health Nursing, Himalayan College of Nursing, Dehradun, IND; 2 Nursing, Himalayan College of Nursing, Dehradun, IND; 3 Child Health Nursing, Swami Rama Himalayan University, Dehradun, IND

**Keywords:** care of preterm babies, knowledge and practices, mother, quasi-experiment, teaching

## Abstract

Background: Preterm birth is a critical global health concern affecting millions of infants annually. Mothers of preterm babies play a pivotal role in their care but often lack the requisite knowledge and skills to provide optimal support. The study aimed to determine the status of mothers’ knowledge and practices regarding the care of preterm babies as well as provide individualized teaching to enhance their performance by improving their knowledge and practice in the care of preterm babies.

Methods: A quasi-experimental equivalent time series design was adopted for this study. A total of 47 mothers of preterm babies were enrolled using the purposive sampling technique. Data were collected using pretested tools such as structured questionnaires on knowledge and practices across various domains of preterm baby care. The data from mothers were collected three times at regular intervals of three days. Each assessment was followed by re-education on preterm baby care.

Results: Most of the mothers (48.9%) were between the age group of 25-30 years and educated up to graduate and above (51.1%). About 87.2% of the mothers had no prior knowledge about preterm baby care. Most of the babies (76.59%) were born between 32 and 37 weeks of gestation. About 63.8% were male babies and weighed between 1500 g and <2500 g (74.47%). Most babies (89.4%) suffered from conditions such as hypoglycemia, hyperbilirubinemia, and respiratory distress syndrome. At baseline, 95.7% of the mothers had average knowledge, which subsequently improved to 100% after teaching preterm baby care (p<0.001). Similarly, 87.23% of mothers had average practices, which improved to a good level (100%) by the final post-test (p<0.001).

Conclusion: Every new mother's knowledge regarding preterm baby care should be assessed. It is very important to teach mothers about the care of preterm babies to empower them to take care of their babies independently. Regular education of mothers significantly improves their knowledge and practices in preterm baby care.

## Introduction

Neonatal health is determined by gestational age and birth method [1]. Babies born before the completion of 37 weeks of gestation are considered premature. Premature babies may have physical characteristics like small size, larger head, scrawny appearance, lanugo hair, higher risk of hypothermia, tachypnoea, respiratory distress, and feeding difficulties. They also have neuromuscular characteristics like a larger square window, less arm recoil, and a greater popliteal angle [2]. Premature babies face the risk of major health conditions, including respiratory distress syndrome, infections, feeding difficulties, learning disabilities, hypoglycemia, hyperbilirubinemia, and lung diseases [3]. Preterm death can result from complications related to preterm delivery, such as congenital abnormalities, hypoxia, or sepsis. Common causes include intraventricular hemorrhage, necrotizing enterocolitis, respiratory distress syndrome, and premature readmission to the neonatal intensive care unit (NICU) [4].

Preterm delivery is a global issue affecting newborn mortality rates and health outcomes [5]. Survival chances depend on the place of birth and the expertise of the healthcare providers. Most preterm infants require specialized care in the NICU. Parents face challenges in caring for preterm infants, and they must take the lead in crucial areas like exclusive breastfeeding, thermoregulation, infection control, immunization, and treatment management [6]. Addressing preterm births is crucial to achieving the 2030 Sustainable Development Goals. Research indicates that preterm children face challenges in cognitive, academic, behavioral, executive functioning, and psychological well-being [7]. Preterm delivery frequently impacts parents' mental health, often resulting in elevated levels of anxiety, depression, and stress. Breastfeeding may be hampered in preterm babies due to poor sucking abilities, separation, and stress. Nurses play a crucial role in supporting preterm mothers in-home care [8].

The birth of a preterm baby is often distressing due to physical separation, changes in size and appearance, and alterations in parental roles [9]. Research recommends postponing the baby's first bath, keeping the cord stump clean, changing diapers frequently, and using oil massage for skin care, as recommended by Madhu et al. (2021) [10].

Premature delivery is a significant cause of death in children under five years of age, with 13.4 million neonates delivered preterm globally in 2020, with over one million dying within 24 hours [11]. In 2020, India recorded approximately 3.02 million preterm births, accounting for over 20% of the global total [12]. Uttarakhand's neonatal mortality rate (32.4 per 1,000 live births) is higher than the national average (24.9), with a mortality rate of 46.5 among children under five years of age. Complications of preterm birth, vaccine-preventable diseases, and the lack of low-cost interventions are the main causes of death. Around 2.4 million deaths occur among neonates, with 2.61 due to infectious causes. The primary cause of post-discharge medical complications in preterm infants readmitted to the NICU is often attributed to gaps in maternal knowledge regarding preterm newborn care [13].

The World Health Organization (WHO) emphasizes measures to enhance preterm birth outcomes, encouraging parents to be knowledgeable about important infant-related care issues at home [14]. Awareness of a health condition can prompt early medical attention, prevent additional consequences, and improve adherence to therapy [15]. This research study examines the knowledge and lived experiences of mothers who have had preterm birth as most preterm babies are readmitted with problems such as hypoglycemia, respiratory distress, jaundice, feed intolerance, and sepsis.

## Materials and methods

Research design and modeling

This study focused on 47 mothers of preterm infants admitted to the Himalaya Hospital in Dehradun, Uttarakhand, India. The study adopted a quasi-experimental, balanced time-series design. Informed written consent was obtained from all participants prior to the commencement of the study. This study was approved by the Ethics Committee of Swami Rama Himalayan University (SRHU/HIMS/ETHICS/2022/404).

Inclusion and exclusion criteria

Inclusion criteria involved mothers of preterm infants admitted to the Himalaya Hospital in Uttarakhand who provided informed written consent and were willing to participate in the structured repeated assessments and individualized teaching sessions. In terms of exclusion criteria, the study implicitly excluded mothers of full-term infants, those unwilling or unable to consent, and mothers of preterm infants who were not admitted to the specified hospital where the study was conducted.

Tool validation

Structured tools were used for data collection in the study, including a knowledge questionnaire, a self-reported practice checklist, and an observational practice checklist related to preterm baby care. These tools were categorized across seven essential domains: body temperature maintenance, hygienic care for the eyes, cord, skin, and diapers, feeding practices and sterilization, recognizing danger signs, immunization and infection prevention, and follow-up care. The questionnaires were designed to assess the mothers' knowledge and practices in each of these seven domains to confirm that they could independently manage critical aspects of their baby's care.

The tools were validated by seven experts from relevant fields such as pediatric nursing, neonatology, and obstetrics. Reliability was assessed using the Spearman-Brown prophecy test, Karl Pearson’s correlation, and Cronbach’s alpha, yielding internal consistency scores of 0.8, 0.9, and 0.8, respectively. The scoring for knowledge and practices was categorized into three levels. Knowledge scores were labeled as 'poor' (0-15), 'average' (16-30), and 'good' (31-45). Practice scores followed similar ranges: 'poor' (0-18), 'average' (19-27), and 'good' (28-36). Scores were evaluated at three intervals: pre-intervention, post-intervention 1, and post-intervention 2, showing significant improvement in both knowledge and practice after each teaching intervention.

Pilot testing

A pilot study was conducted with five mothers of premature infants who met the inclusion criteria to determine the safety and feasibility of the research. The study was deemed feasible.

Data collection

**Figure 1:**
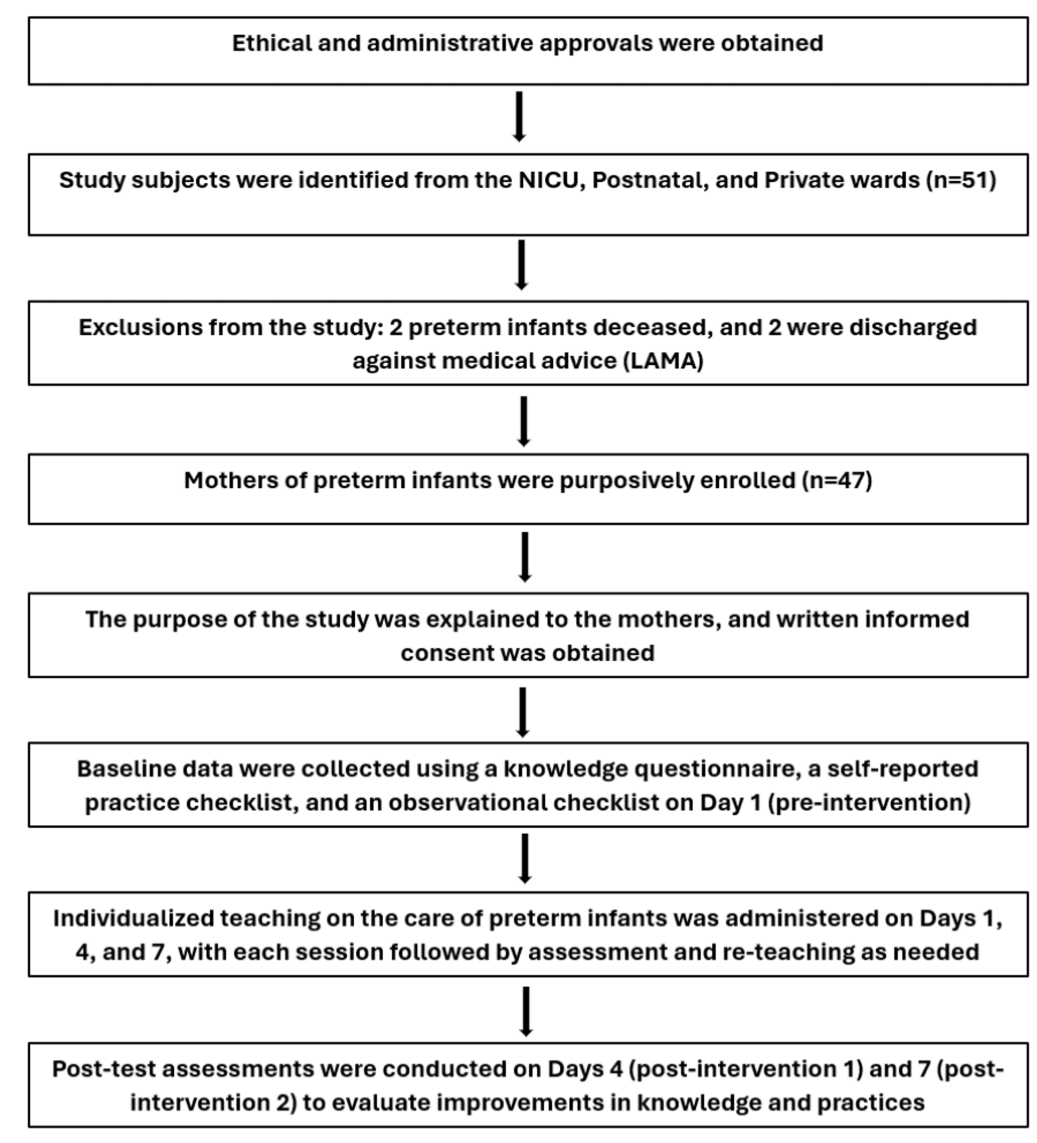
Flow diagram of participant selection, data collection, and individualized education interventions for preterm infant care LAMA: leave against medical advice

Data from 47 mothers of premature infants admitted to Himalaya Hospital, Uttarakhand, were collected. The aim of the study was explained to the mothers at the beginning of the study, and data were collected according to a structured schedule on day 1 (pre-intervention), day 4 (post-intervention 1), and day 7 (post-intervention 2), with a three-day interval between each assessment. Each assessment consisted of evaluating mothers' knowledge and practices in key domains of preterm baby care and then individualized teaching sessions. The researchers were able to measure improvements in the mother's ability to care for their infants, and each step of the intervention was systematically followed. Days 4 and 7 demonstrated assessments and reteaching to help the mothers build the skills and confidence to do critical aspects of their baby's care independently (Figure 1).

Statistical analysis

The data was analyzed statistically using IBM SPSS Statistics for Windows, Version 20 (IBM Corp., Armonk, USA). Demographic and baseline characteristics of participants were summarized with descriptive statistics including means, standard deviations, and percentages. The Kolmogorov-Smirnov test was used to check the normality of the data, and consequently, further statistical methods were selected. Changes in knowledge and practice levels at the three intervention points (pre-intervention, post-intervention 1, and post-intervention 2) were also tested using repeated measures analysis of variance (ANOVA). 

## Results

Most of the mothers (48.9%) were aged between 25 and 30 years. The majority of them (76.6%) were homemakers, belonged to joint families (57.4%), lived in urban areas (66%), and were educated up to graduation (Bachelor's degree) or higher (51.1%). About 87.2% belonged to Hindu families, and 48.94% had a family income between ₹10,000 and ₹30,000. Approximately 61.7% of mothers were primigravida with no history of illness (97.9%) or complications (93.6%) during delivery. Additionally, 87.2% of the mothers had no prior information regarding the care of preterm babies. Most of the babies (76.59%) were born between 32 and <37 weeks of gestation. Around 63.8% were male, and 74.47% weighed between 1500 and <2500 grams. About 72.3% of the babies were delivered via lower segment cesarean section (LSCS), and all deliveries occurred in hospitals. No complications (91.5%) were found in the babies during delivery. However, 89.4% of the children became ill after delivery, with 28.5% of these cases involving hyperbilirubinemia and respiratory distress syndrome (RDS), as shown in Table 1.

**Table 1:** Frequency and percentage distribution of baseline information of mother and baby (N=47) VLBW: very low birth weight; LBW: low birth weight; RDS: respiratory distress syndrome

S. No.	Variable	Frequency (f)	Percentage (%)
1	Age		
	a) 21-25 years	7	14.90
	b) 25-30 years	23	48.90
	c) 31-35 years	15	31.90
	d) 36-40 years	2	4.30
2	Education status		
	a) No formal education	1	2.10
	b) Primary education	4	8.50
	c) Secondary education	18	38.30
	d) Bachelor's degree and above	24	51.10
3	Occupation		
	a) Employed	11	23.40
	b) Homemaker	36	76.60
4	Religion		
	a) Hindu	41	87.20
	b) Muslim	5	10.60
	c) Sikh	1	2.10
5	Monthly family income (Rupees)		
	a) Up to 10000	5	10.64
	b) >10000-30000	23	48.94
	c) >30000-60000	14	29.79
	d) >60000-100000	5	10.63
6	Type of family		
	a) Nuclear	20	42.60
	b) Extended	27	57.40
7	Residential Area		
	a) Rural	7	14.90
	b) Urban	31	66.00
	c) Semi-urban	9	19.10
8	Gravida		
	a) Primi	29	61.70
	b) Multi	18	38.30
9	Medical history		
	a) Yes (viral infection)	1	2.10
	b) No	46	97.90
10	Any delivery complications		
	a) Yes	3	6.40
	b) No	44	93.60
10 a	Types of delivery complications (n=3)		
	a) Pre-eclampsia	2	66.60
	b) Premature rupture of membranes (PROM)	1	33.30
11	Any previous information regarding preterm care		
	a) Yes	6	12.80
	b) No	41	87.20
11 a	Source of information (n=6)		
	a) Previous experience	4	66.60
	b) Multimedia	2	33.30
12	Baby’s profile		
12.1	Gestational week		
	a) <28 weeks (extremely preterm)	1	2.13
	b) 28-<32 weeks (very preterm)	10	21.28
	c) 32-<37 weeks (moderate to late preterm)	36	76.59
12.2	Gender of baby		
	a) Male	30	63.80
	b) Female	17	36.20
12.3	Birth weight of the baby		
	a) 1000-<1500g (VLBW)	12	25.53
	b) 1500-<2500g (LBW)	35	74.47
12.4	Birth order of baby		
	a) 1st	29	61.70
	b) 2nd	16	34.00
	c) 3rd	2	4.30
12.5	Any complication at birth		
	a) Yes	4	8.50
	b) No	43	91.50
12.5 a	Complications at birth (n=4)		
	a) Meconium aspiration	2	50.00
	b) Birth asphyxia	1	25.00
	c) Respiratory distress	1	25.00
12.6	Mode of delivery		
	a) Normal delivery	13	27.70
	b) Lower segment cesarean section (LSCS)	34	72.30
12.7	Place of delivery		
	a) Hospital	47	100.00
12.8	Any illness of the child post-delivery		
	a) Yes	42	89.40
	b) No	5	10.60
12.8 a	Illness of the child post-delivery (n=42)		
	a) Hypoglycemia	9	21.40
	b) Hyperbilirubinemia	11	26.19
	c) Respiratory distress	10	23.80
	d) Hyperbilirubinemia with RDS	12	28.50

Assessment of knowledge

Through the structured pre-test assessment, 95.7% of the mothers were classified as having an 'average' knowledge level regarding preterm baby care. Specifically, 44.6% of mothers demonstrated general knowledge about preterm babies, 33.42% were aware of body temperature maintenance, 49.85% knew about danger signs, 39.25% about immunization and infection prevention, and 47.2% recognized the importance of regular follow-up care for preterm infants. After the educational intervention, the post-test scores showed that knowledge improved to 100% in all assessed domains, as indicated in Table 2. Knowledge levels were categorized based on score ranges, which outline the criteria for each level.

**Table 2:** Mothers' knowledge and practice levels regarding care of preterm babies (N=47)

Variable	Category	Score range	Pre-test		Post-test 1 (Day 4)		Post-test 2 (Day 7)	
			f	%	f	%	f	%
Knowledge	Poor	0-15	1	2.12	0	0	0	0
	Average	16-35	45	95.70	1	2.12	0	0
	Good	36-45	1	2.12	46	97.80	47	100
Practice	Poor	0-18	1	2.13	0	0	0	0
	Average	19-27	41	87.23	16	34.04	0	0
	Good	28-36	5	10.64	31	65.96	47	100

Knowledge scores of 47 mothers regarding the care of preterm infants were measured at three key intervals: pre-intervention, post-intervention 1, and post-intervention 2. Scores were categorized into 'poor', 'average', and 'good’ based on predefined score ranges rather than averaging. The results showed statistically significant improvements across these categories at each assessment point as verified by repeated measures ANOVA (F=1075.5, p<0.001). By the final assessment, 100% of mothers had achieved the 'good' category in knowledge across all domains, reflecting the effectiveness of the structured interventions.

The improvement observed in the final assessment was higher than the pre-test knowledge scores in all domains, including general care of preterm babies, maintenance of body temperature, eye, cord, skin, and diaper care, feeding care, sterilization of feeding equipment, danger signs, immunization and sepsis prevention, and follow-up care, as detailed in Table 3.

**Table 3:** Domain-wise improvement of knowledge of mothers after individualized teaching on care of preterm babies (N=47)

Domains	Max score	Range	Mean ± SD			Average knowledge score (%)		
			Pre-test	Post-test 1	Post-test 2	Pre-test	Post-test 1	Post-test 2
General information on preterm baby	6	0-6	2.68 ± 1.18	5.04 ± 0.62	5.87 ± 0.33	44.60	84	97.83
Maintenance of body temperature	7	0-7	2.34 ± 1.16	5.64 ± 0.96	6.8 ± 0.39	33.42	80.57	97.14
Eye, cord, skin, and diaper care	11	2-11	6.94 ± 1.20	8.83 ± 0.98	10.6 ± 0.59	63.09	80.27	96.36
Feeding care and sterilization of feeding equipment	5	1-5	2.68 ± 0.69	4.02 ± 0.67	4.89 ± 0.312	53.60	80.40	97.80
Danger signs	7	1-7	3.49 ± 0.99	4.96 ± 1.02	6.64 ± 0.48	49.85	70.85	94.85
Immunization and prevention of infection	4	0-4	1.57 ± 0.97	3.04 ± 0.55	3.89 ± 0.31	39.25	76	97.25
Follow-up care	5	1-5	2.36 ± 0.98	3.85 ± 0.80	4.96 ± 0.20	47.20	77	99.20

Assessment of practice

**Figure 2:**
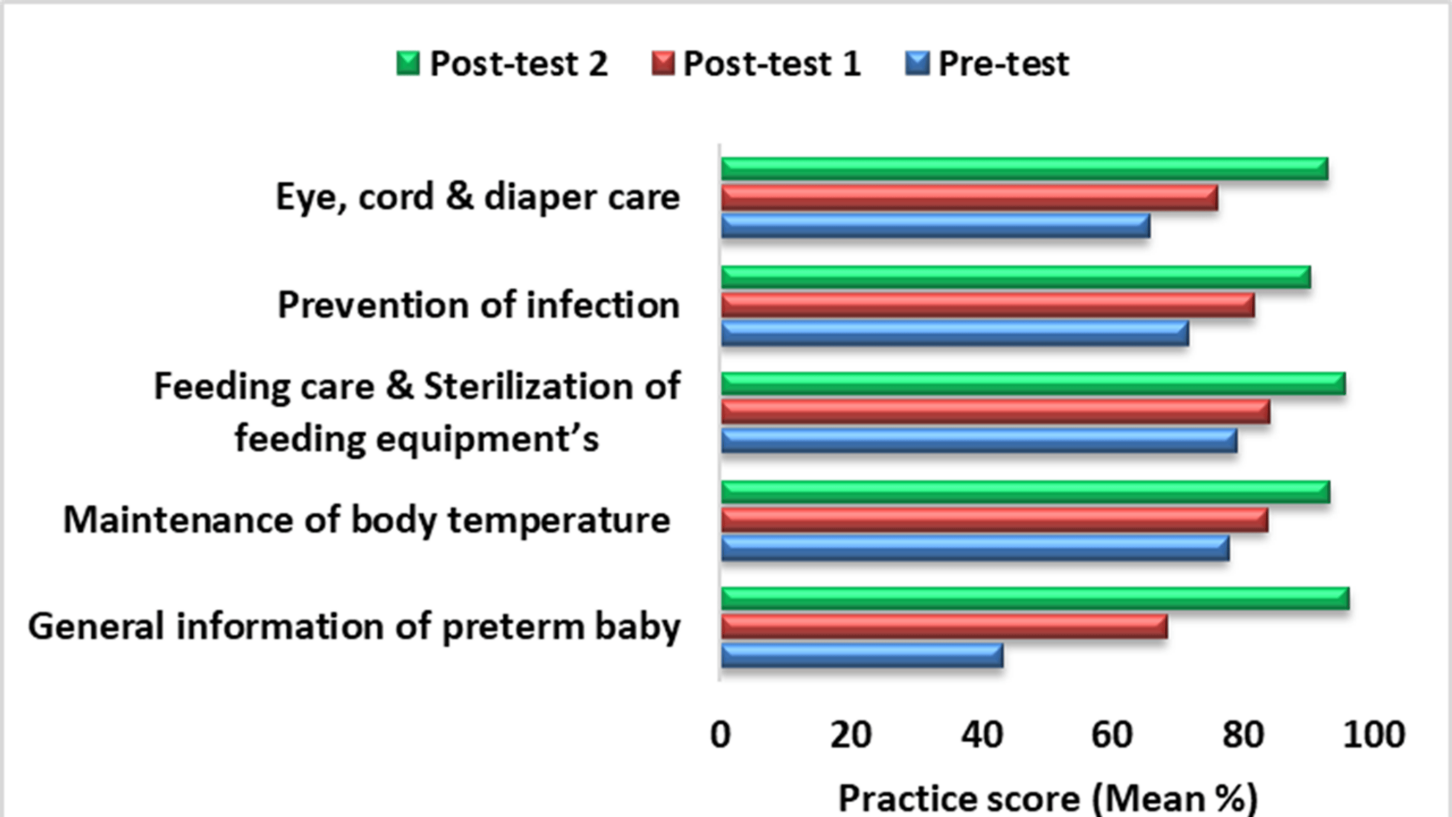
Domain-wise distribution of mean percentage of practice score of mothers of preterm babies

Initially, most of the mothers were aware of and practiced changing wet diapers immediately (80.8%). However, they did not use appropriate techniques while providing care to their preterm infants, particularly in areas such as hand washing, kangaroo mother care (KMC), breastfeeding techniques, umbilical cord care, eye care, and infection prevention, as detailed in Tables 5-6. Gradual improvement was observed in maintaining body temperature (93.3%), feeding care and sterilization (95.6%), cord and eye care (93%), and infection prevention (90.3%), with significant results (p<0.001), as shown in Tables 5-6 and Figure 2. Initially, 87.23% of the mothers had an average level of practice regarding preterm baby care, but they achieved a 100% good practice level by the final assessment (p<0.001), as indicated in Table 4. Surprisingly, no significant improvement was seen in hand washing (46.8%), eye care (40.4%), and umbilical cord care (29.7%). Additionally, the mothers' practice of directly kissing the faces of preterm newborns did not change significantly, from 95.7% to 93.6%, as shown in Table 6. Furthermore, a moderate correlation was found between the mothers' knowledge and practice at the end of the training, with a correlation coefficient of r=0.40.

**Table 4:** Improvement of practice scores of mothers after individualized teaching on care of preterm (N=47) df=2 (5.99), *significant, p<0.05 level, repeated measure analysis of variance (ANOVA)

Variable		Pre-test	Post-test 1	Post-test 2	F value	P value
		Mean ± SD	Mean ± SD	Mean ± SD		
Practice	Self-reported practice	24.28 ± 2.73	28.28 ± 2.20	33.64 ± 1.37	273.24*	p<0.001
	Observational checklist	3.11 ± 1.08	5.64 ± 0.64	7.98 ± 0.76	462.8*	p<0.001

**Table 5:** Domain-wise improvement of the practice of mothers after individualized teaching on care of preterm babies (N=47)

Domains	Max score	Range	Mean ± SD			Average knowledge score (%)		
			Pre-test	Post-test 1	Post-test 2	Pre-test	Post-test 1	Post-test 2
General information on preterm baby	6	0-6	2.60 ± 1.280	4.09 ± 0.905	5.77 ± 0.598	43.30	68.16	96.16
Maintenance of body temperature	6	0-6	4.66 ± 1.089	5.02 ± 0.847	5.60 ± 0.742	77.60	83.60	93.30
Feeding care and sterilization of feeding equipment	6	3-6	4.74 ± 0.765	5.04 ± 0.779	5.74 ± 0.530	79	84	95.60
Prevention of infection	8	4-8	5.72 ± 1.210	6.53 ± 1.039	7.23 ± 0.758	71.50	81.60	90.37
Eye, cord, and diaper care	10	4-10	6.55 ± 1.265	7.60 ± 1.097	9.30 ± 0.858	65.50	76	93

**Table 6:** Observed practices of care of preterm babies by mothers "f" values represent the frequency (the number of mothers who reported following a specific practice)

Practices followed by mothers	Pre-test		Post-test 1		Post-test 2	
	f	%	f	%	f	%
Washed hands before touching the baby	1	2.12	7	14.80	22	46.80
Warmed the hands before touching the baby	7	14.80	28	59.50	46	97.80
Provide effective kangaroo mother care	10	21.27	37	78.70	46	97.80
Breastfeed in the correct position	9	19.14	38	80.80	43	91.40
Burped the baby after feed	17	36.17	29	61.70	47	100
Check the umbilical cord	1	2.12	3	6.38	14	29.70
Kissed on the baby’s face	45	95.70	44	93.60	44	93.60
Changed wet diaper immediately	38	80.80	45	95.70	47	100
Checked baby’s eyes for any discharge	1	2.12	3	6.38	19	40.40
Keep the umbilical cord outside the diaper	17	36.17	31	65.90	47	100

## Discussion

This study highlights the critically important role of targeted educational interventions in improving maternal knowledge and practices of preterm infant care. While 51.1% of mothers had a graduate or higher education level, initial assessments showed that 95.7% of mothers had only moderate knowledge of preterm care, indicating that general education does not guarantee specific competencies in neonatal health. The post-intervention improvements were significant in infection prevention, temperature control, and hygiene practices, which is consistent with other research findings that structured, topic-specific education significantly improves caregivers’ knowledge and practices [16].

One of the key strengths of this study was the structure of the intervention, which enabled the measurement of knowledge and practice improvements in multi-domains of preterm care. Reliable data collection was also ensured through the use of validated assessment tools, and furthermore, the study was strongly able to measure intervention outcomes. Preterm care infection prevention was greatly improved post-intervention. Only 2.12% of mothers first practiced proper handwashing, but after the intervention, assessments showed infection prevention knowledge at 97.25% and practice adherence at 90.37%. Bacterial infections, such as sepsis, pneumonia, and meningitis, are major causes of death in preterm infants and significantly contribute to the infant mortality rate in India [17]. Cultural factors such as direct contact (e.g., kissing the baby), however, were not amenable to change and showed only minimal improvement. These findings are consistent with work that underscores the importance of repeated culturally sensitive interventions to break entrenched behavior [18].

This study also highlights one of the critical areas, the importance of early and adequate maternal knowledge about preterm care, specifically the warning signs and preventive measures. Delayed early care has been identified as a leading cause of infant mortality and so it is important for mothers to recognize and respond to danger signs. The WHO defines these danger signs as refusal to eat, abnormal temperature, jaundice, and localized infection signs among others [19]. As part of this study, mothers were educated on identifying symptoms of jaundice, dehydration, difficulty breathing, and the causes of diarrhea in preterm infants in order to help empower mothers to be proactive in the care of their preterm infants.

Another important aspect of maternal education included in this intervention was knowledge about vaccination. Only 39.25% of mothers initially understood the significance of neonatal immunizations, such as polio and Bacillus Calmette-Guérin (BCG) vaccines. According to the WHO data, timely vaccination can save the lives of about 2.5 million babies annually, and education of this kind remains critical [20,21]. Maternal knowledge of vaccination increased post-intervention and practice scores for neonatal immunization reached 90.37%. This improvement is consistent with previous research that structured, targeted education increases maternal capacity to contribute to infant health and decreases risks for preventable diseases [22].

Another area of limited baseline knowledge was temperature maintenance, including KMC. Initially, only 33.42% of mothers understood the importance of temperature regulation in preventing hypothermia, a condition to which preterm infants are especially vulnerable. Adherence to KMC improved to 97.8% after the intervention, consistent with other studies demonstrating that education on KMC improves both temperature regulation and early breastfeeding initiation in preterm infants [23]. However, despite these gains, specific practices such as warming hands before touching the infant did not show much adherence, indicating that repeated instruction is needed to reinforce such actions [24].

However, even with these improvements, care practices such as eye care and proper umbilical cord care proved challenging, with adherence rates of only 40.4% and 29.7% after the intervention. This study found that complex neonatal care tasks typically require hands-on training and continuous support to achieve consistency in caregiver practices. Repeated guidance and on-site demonstrations may therefore be necessary to achieve full adherence to these more complex practices.

Limitations of the study 

This study was limited to a single hospital, so the results may not generalize to other settings. Additionally, the sample size was particularly small (47 participants) and the duration of the intervention was short, allowing only immediate post-intervention effects to be evaluated. Sustained improvements in maternal knowledge and practice would need to be observed over time and longer-term studies would be required to establish whether these improvements are maintained. Although the quasi-experimental design and specific intervention setting also limit external validity, the results may not be generalized to the wider healthcare environment without further validation. Future research should investigate the scalability and effectiveness of individualized educational interventions across diverse contexts, addressing context-dependent needs in larger, multi-center studies.

## Conclusions

Parents are the primary caregivers of preterm babies and, as such, must be equipped with the knowledge to provide proper care. Assessing mothers’ understanding and educating them on how to care for preterm infants is crucial in promoting a culture of independent maternal care. In this study, teaching mothers helped improve their knowledge and practices related to home care for preterm babies. The mothers gained a better understanding of general preterm care, temperature regulation, hygiene, feeding, recognizing signs of illness, infection prevention, immunization, and follow-up care. Regular educational interventions assist mothers in appreciating the importance of hygiene in promoting the well-being of preterm infants. To ensure the validity of such research, it should be conducted over a longer period with a larger and more diverse participant group. Identifying areas where mothers lack knowledge and increasing their understanding of preterm care enables them to address their babies' individual needs, thereby improving the health outcomes of preterm infants.
